# A Lethal Manifestation of Chronic Active Epstein-Barr Virus Infection: A Case Report

**DOI:** 10.7759/cureus.30158

**Published:** 2022-10-10

**Authors:** Yousef Alotaibi, Mahdi Albogami, Abdulrahman Alsaedy, Rashed Khubrani, Bushra Al Ahmadi

**Affiliations:** 1 College of Medicine, King Saud Bin Abdulaziz University for Health Sciences, Riyadh, SAU; 2 Department of Infectious Diseases, King Abdulaziz Medical City, Riyadh, SAU; 3 Department of Pathology and Laboratory Medicine, King Abdulaziz Medical City, Riyadh, SAU

**Keywords:** infectious disease, standards of diagnosis, b cells/nk lymphocytosis, chronic active epstein-barr virus (caebv), epstein-barr virus (ebv)

## Abstract

Chronic active Epstein-Barr virus infection (CAEBV) is a rare and lethal condition caused by persistent Epstein-Barr virus (EBV) infection. Signs and symptoms of CAEBV infection include fever, lymphadenopathy, and hepatosplenomegaly.

Due to life-threatening consequences such as multiple organ failure, hemophagocytic syndrome, EBV-positive lymphoproliferative illness, and coagulopathy, early identification is important for successful therapy. However, because of the wide range of clinical symptoms, it might be difficult to diagnose the disease due to limited clinical experience and a low number of reports.

We report a case of CAEBV in a 59-year-old woman from Saudi Arabia. We present the hospital course of the patient from admission until the patient's death as well as the clinical and pathological findings with a review of the literature. This is a rare case of CAEBV in Saudi Arabia.

## Introduction

Epstein-Barr virus (EBV) is a member of the herpes virus family, and it is known as human herpesvirus 4. More than 90% of the world's population carries EBV as a life-long, latent infection of B lymphocytes [1]. EBV can spread through blood and semen during sexual contact and blood transfusions, but most commonly, it spreads through saliva [2]. EBV infection occurs usually in childhood, but EBV infection presented during adolescence often results in infectious mononucleosis.

Infectious mononucleosis is caused by primary EBV infection and occurs in only a small number of infected individuals. The disease is characterized by lymphocyte proliferation due to the clonal expansion of reactive T cells against EBV-infected B cells [3]. Most patients are between 15 and 40 years old. The patient may experience lethargy, malaise, headache, stiff neck, and dry cough. In established disease, the following features may be found: lymphadenopathy, pharyngitis, mild or severe fever of unknown origin, hepatosplenomegaly with or without jaundice, severe headache, ophthalmic signs such as photophobia, conjunctivitis, and periorbital edema, and pancytopenia [3].

Only a few individuals infected with EBV develop a life-threatening condition termed chronic active EBV disease (CAEBV) [4-7]. In Western countries, CAEBV infection is rare. Asians and Native Americans, on the other hand, have a higher incidence, with children and adolescents being more affected. In Asia, the mean age at which CAEBV occurred was 11.3 ranging from nine months to 53 years [8]. More serious complications of CAEBV infection may include anemia, neuropathy, liver failure, or interstitial pneumonia. CAEBV occurs when the symptoms of the infection are not resolved, and the virus remains active. Symptoms are intermittently present or constant and tend to get worse with progression. It is diagnosed symptomatically, with clinical examination and serologic tests that show EBV DNA [9-11].

We report a case of CAEBV in a 59-year-old Saudi woman in this article. We describe the course of the disease from admission until the patient’s death.

## Case presentation

We present a 59-year-old patient who presented to the emergency room (ER) with a fever of unknown origin and vague abdominal pain. On admission, the patient's temperature was 37.9°C, the blood pressure was 115/72 mmHg, and the respiration rate was 21/min. Laboratory data showed hemoglobin (Hb) of 82 g/L, platelet of 184 × 109/L, and white blood cells (WBC) of 2.40 x 10^9^/L with the differential count of segmented neutrophils 77.5%, lymphocytes 9%, and monocytes 4.5%. The liver function test (LFT) revealed aspartate aminotransferase (AST) of 134 U/L, alanine aminotransferase (ALT) of 76 U/L, and alkaline phosphatase (ALP) of 1,673 U/L. Renal function test (RFT) results were as follows: estimated glomerular filtration rater (eGFR) of 139 mL/min/1.73 m^2^, blood urea nitrogen (BUN) of 4.8 mmol/L, and creatinine of 43 µmol/L. Coagulation studies showed increased partial thromboplastin time (PTT) of 38.6 seconds and an international normalized ratio (INR) of 1.03. The results of the serologic test were EBV-IgG (+), EBV-IgM (-), EBV nuclear antigen (EBNA) (+), and EBV-EA (+). A peripheral blood smear showed normocytic and normochromic red blood cells (RBCs) with mild leukopenia. Bone marrow aspirate showed moderate cellularity with a limited number of bony spicules along with increased eosinophils. Polymerase chain reaction (PCR) analysis for tuberculosis was done, which was negative, and EBV DNA was detected.

A computed tomography (CT) scan of the abdomen revealed an enlarged spleen that was 14.2 cm in length with multiple hypodense lesions, and hepatic dimension measured 25 cm craniocaudally. Evidence of abdominal lymphadenopathy was found.

On the first day of admission, she was admitted to the ICU due to severe sepsis, metabolic lactic acidosis, and hyperkalemia. She received anti-hyperkalemic measures. On the 15th day of admission, the hematologist reviewed the case. Since the multiple biopsies of various sites did not reveal any diagnosis, a diagnostic splenectomy was suggested to reach a diagnosis. The splenectomy was planned after one week because optimization of the patient's liver function test was required before the surgery.

On the 23rd day of admission, the patient clinically deteriorated to a critical condition and presented with hypotension, tachycardia, and high-grade fever (38.6℃). The Hgb was 61 g/L and platelets were 22 × 10^9^/L; thereupon 1.5 L of normal saline was administered intravenously. Her condition deteriorated into sepsis and septic shock; the patient was given vancomycin, meropenem, caspofungin, and ganciclovir intravenously. Further deleterious manifestations included multiple organ dysfunction; the LFT and RFT were as follows: AST 708 U/L, ALT 155 U/L, ALP 1,111 U/L, eGFR 37 mL/min/1.73 m^2^, BUN 17.4 mmol/L, and creatinine 135 µmol/L. Furthermore, the patient had lactic acidosis and disseminated intravenous coagulopathy (DIC); coagulation tests showed worsened PTT > 177 s, INR 79, fibrinogen < 0.30 g/L, and D-dimer 1.10 mg/L. These were attributed to liver failure or severe sepsis since there was no other obvious source clinically. She has been comatosed off sedation with no brain stem reflexes with overall mottled skin, crackles heard on auscultation, pitting generalized edema, and oozing blood from puncture sites. On the next day, the patient developed a decrease in urine output and acidosis with severe shock, and the patient was unstable to tolerate continuous venovenous hemodialysis (CVVHD).

On the 25th day, while the patient was in the ICU, the patient had refractory sepsis and DIC despite being treated with antibiotics, antiviral, and antifungal. The patient was managed on maximum life support measures with mechanical ventilation, vasopressors, and frequent blood transfusions to correct her coagulopathy. Additionally, sodium bicarbonate infusion and anti-hyperkalemic were given intravenously since the patient was unstable hemodynamically, and dialysis was contraindicated. The patient has expired because of multiorgan failure and DIC due to septic shock.

## Discussion

**Figure 1:**
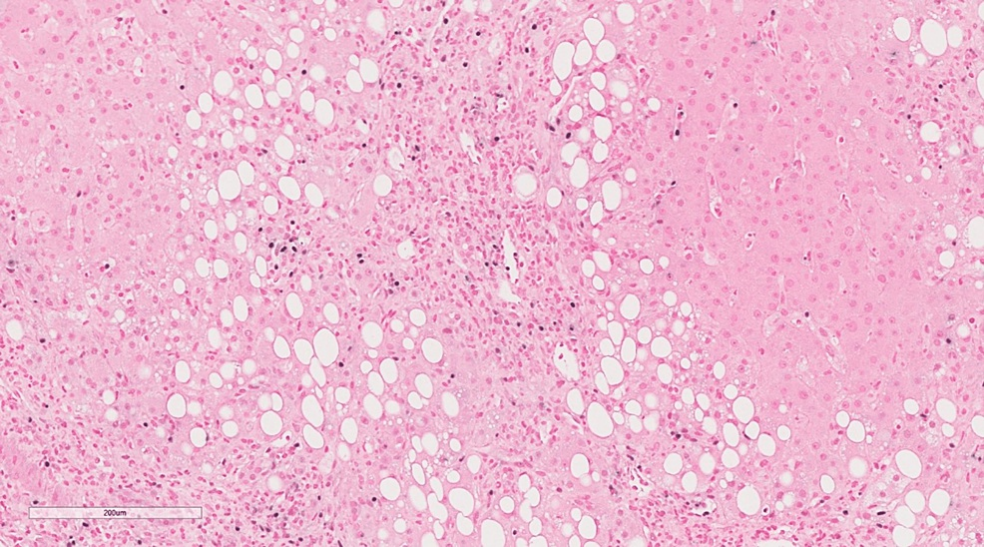
In situ hybridization for EBV showing multiple positive lymphocytes (IHC original magnification x 200) IHC: Immunohistochemical staining; EBV: Epstein-Barr virus.

**Figure 2:**
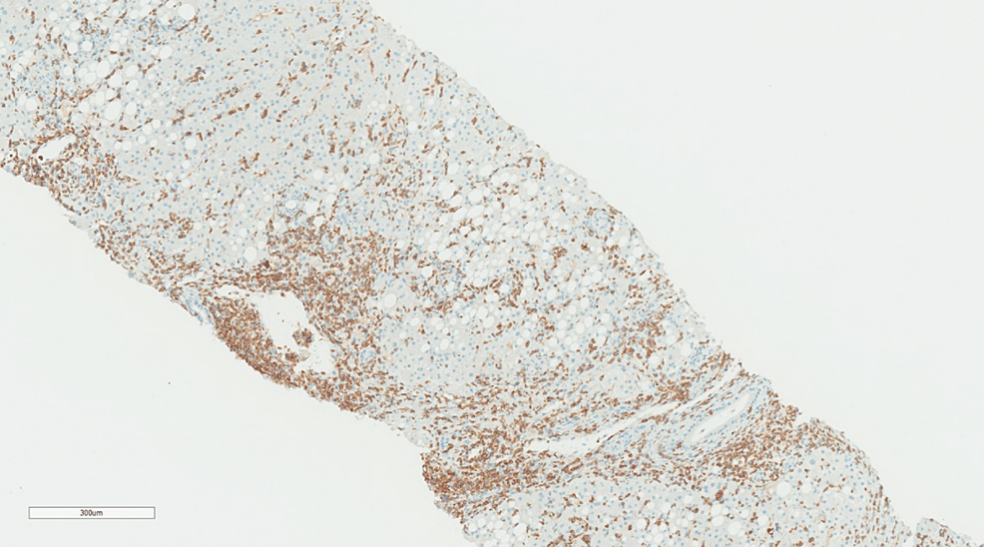
Immunohistochemistry of CD3 cells showing most of the infiltration by T lymphocytes (IHC original magnification x 300) IHC: Immunohistochemical staining.

EBV infection in adolescents and young adults often results in infectious mononucleosis. The EBV pathogenesis is characterized by the infection of B cells by binding with the EBV receptor on the surface of the cell. CAEBV, on the other hand, is a rare complication of the infection and is a progressive EBV infection of T and natural killer (T/NK) cells with an increase of the EBV antibody titers over a long-term interval [12,13]. In this case, in situ hybridization showed multiple positive lymphocytes (Figure 1). Immunohistopathology of CD3 revealed most of the infiltration by T lymphocytes (Figure 2).

A criterion for CAEBV infection was proposed by Straus et al. in 1988. The criteria included histologic evidence of major organ involvement, high abnormal antibody titers of EBV, high quantities of EBV in tissues, and illness lasting more than six months [14]. Okano et al. reviewed the clinical, pathological, and virological findings of 26 patients in the English-language literature and their 10 cases (eight from Japan and two from the United States) as well using the criteria of Straus et al. Some of the cases showed that all of the patients had exceptionally elevated antibody titers against the EBV, even though some patients demonstrated a length of sickness of less than six months, which does not meet the criteria stated by Straus et al. [4]. Ishihara et al. in 1995 described the findings of 39 children with SCAEBV in Japan. In the study, high anti-EBV antibody titers were present in 57.1% of anti-EA IgG, 47.1% were anti-EA IgA-positive, 28.2% for anti-viral capsid antigen (anti-VCA) IgG, and 72.7% were anti-VCA IgA [15].

**Figure 3:**
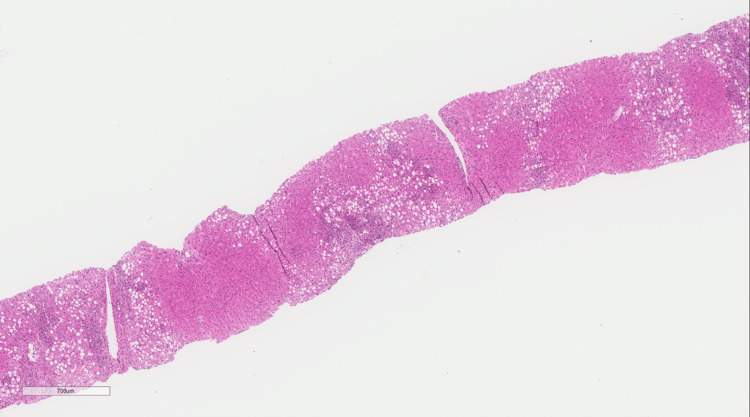
Low power of liver tissue shows vague nodularity with moderate steatosis primarily affecting Zone 3 and extending to Zone 2 as well as some amount of inflammation involving the portal tracts (H&E original magnification x 100) H&E: Hematoxylin and eosin.

**Figure 4:**
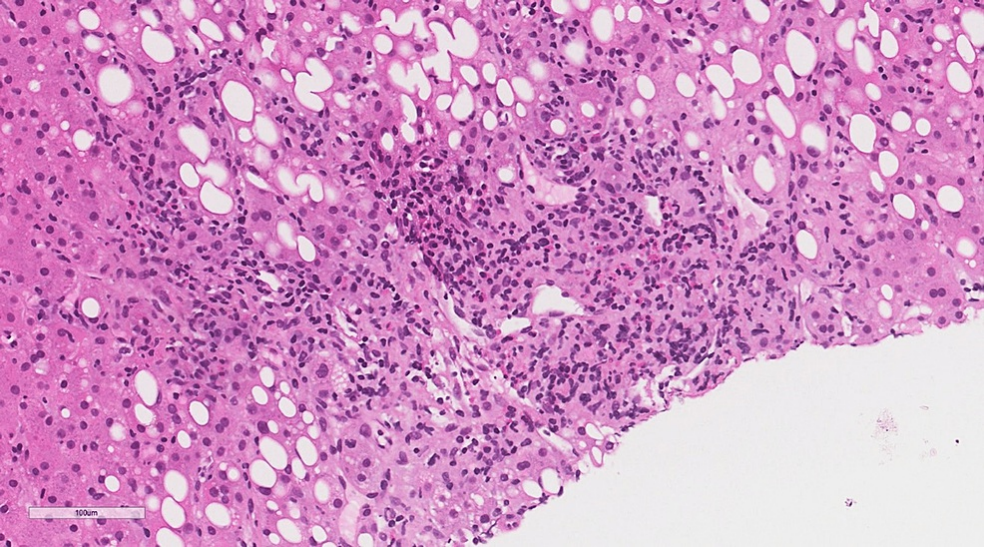
Higher power on portal tracts shows portal and periportal lobular inflammation with the presence of a large number of granulomas, most of which qualify for the morphological features of lipogranulomas (presence of fat vacuoles surrounded by epithelioid histiocytes and some eosinophils). Some granulomas show numerous eosinophils (H&E original magnification x 400). H&E: Hematoxylin and eosin.

In immunocompetent patients, it is easy to distinguish acute from past infection of EBV using only three parameters (VCA IgG, VCA IgM, and EBNA). If the patient has positive VCA IgG and VCA IgM with negative EBNA, it indicates acute infection, whereas positive VAC IgG and EBNA with negative VCA IgM suggest past infection [16]. In our patient, a quantitative analysis of anti-EBV viral load quantitated by real-time PCR was performed, and it showed a viral load of 156,400 copies/MI. The results of the serologic test were EBV-IgG (+), EBV-IgM (-), EBNA (+), and EBV-EA (+). Pathologic examination identified predominant T-cell lymphocytic infiltration in the portal, periportal area, and sinusoids with focal infiltration of venous and arteriolar walls (Figures 3, 4). The patient had a fever, hepatosplenomegaly, and multiple enlarged cervical lymph nodes. The clinical course of this patient did not meet the criteria suggested in the previous studies, in which the supposed duration was six months. In our case, it did not last more than four months from the start of the disease to death [4]. However, few patients showed typical clinical and laboratory findings in the previous studies who died of the disease less than three months after the disease started. Thus, our present case points out that based on the constellation of clinical and laboratory findings, the diagnosis of CAEBV should be considered and not be limited by disease duration criteria.

CAEBV has several characteristics. The fact that T or NK cells' cytotoxic activity is diminished shows that the chronic pre-occupation of the cells to combat the active infection could lead to a form of acquired immunodeficiency [11]. Asians or people from Central or South America have a higher risk of the disease, showing that genetics may play a role in the disease process. According to a recent comprehensive genomic investigation using whole-exome sequencing, CAEBV germline alterations are rare, but somatic driver mutations are common in EBV-infected cells[11].

## Conclusions

In conclusion, CAEBV disease is a rare acute sequela of a long-term infection that preludes to a potentially lethal attack on the human biological systems. The clinical awareness of this disease in patients with nonspecific symptoms and systemic manifestation, as well as a multidisciplinary diagnostic strategy to establish a diagnosis, is important.
